# Fine construction of gene coexpression network analysis using GTOM and RECODE detected a critical module of neuroblastoma stages 4 and 4S

**DOI:** 10.1186/s41065-024-00342-y

**Published:** 2024-11-14

**Authors:** Fumihiko Nakamura, Yushi Nakano, Shiro Yamada

**Affiliations:** 1 Faculty of Engineering, Kitami Institute of Technology, 165, Koen-cho, Hokkaido 090-8507 Japan; 2 Department of Mathematics, Hokkaido University, Kita 10, Nishi 8, Kita-ku, Sapporo, Hokkaido 060-0810 Japan; 3 Department of Pediatrics, Usui Hospital, 1-9-10 Haraichi, Annaka, Gunma 379-0133 Japan

**Keywords:** Neuroblastoma, Gene coexpression network analysis, Generalized topological overlap measure, RECODE

## Abstract

**Background:**

Stage 4 neuroblastoma (NBL), a solid tumor of childhood, has a poor prognosis. Despite intensive molecular genetic studies, no targetable gene abnormalities have been identified. Stage 4S NBL has a characteristic of spontaneous regression, and elucidation of the mechanistic differences between stages 4 and 4S may improve treatment. Conventional NBL studies have mainly focused on the detection of abnormalities in individual genes and have rarely examined abnormalities in gene networks. While the gene coexpression network is expected to contribute to the detection of network abnormalities, the fragility of the network due to data noise and the extraction of arbitrary topological structures for the high-dimensional network are issues.

**Results:**

The present paper concerns the classification method of stages 4 and 4S NBL patients using highly accurate gene coexpression network analysis based on RNA-sequencing data of transcription factors (TFs). In particular, after applying a noise reduction method RECODE, generalized topological overlapping measure (GTOM), which weighs the connections of nodes in the network structure, succeeded in extracting a cluster of TFs that showed high classification performance for stages 4 and 4S. In addition, we investigated how these clusters correspond to clinical information and to TFs which control the normal adrenal tissue and NBL characters.

**Conclusions:**

A clustering method is presented for finding intermediate-scale clusters of TFs that give considerable separation performance for distinguishing between stages 4 and 4S. It is suggested that this method is useful as a way to extract factors that contribute to the separation of groups from multiple pieces of information such as gene expression levels.

**Supplementary Information:**

The online version contains supplementary material available at 10.1186/s41065-024-00342-y.

## Background

Neuroblastoma (NBL) is a pediatric malignancy with one of the worst prognoses. Although there have been many studies of NBL [1–5], the specific genetic abnormalities that may be exploited for targeted therapy have not been discovered, except in specific cases, such as ALK mutations. In general, pediatric solid tumors, such as NBL, have fewer genetic mutations compared with other malignancies; however, many chromosomal deletions and duplications (segmental chromosomal aberrations) occur, which indicates abnormalities in the genomic system.

Stage 4 (st4) NBLs with metastasis tend to be resistant to chemotherapy; however, there is a unique type of st4 NBL with a good prognosis, known as “special stage 4” (4S). It has several characteristics, such as an age at diagnosis of 1.5 years and less, and metastatic sites that are limited to the liver, skin, and bone marrow. Of these, the most interesting feature is that it spontaneously regresses [2, 6]. Therefore, identifying genetic and genomic differences between st4 and 4S may lead to the development of new treatments, such as regression induction therapy [7].

Whole exome sequencing could not reveal recurrent driver gene mutations in half of the NBL tumors [1, 8], and previous papers reporting on the characteristics of 4S did not agree on the causative gene expressions [9–11]. These findings indicate that it is difficult to elucidate the mechanism of tumorigenesis and regression mediated by NBL via the current methods, which rely heavily on identifying genetic abnormalities.

Therefore, we conducted a gene coexpression network (GCN) analysis to analyze the genomic system of NBL stages 4 and 4S. Usually, when comparing two different tissues or conditions, a gene expression analysis, such as DESeq2, is performed. However, this method compares differences in a single gene between diseased or healthy tissues, and important information regarding the gene system (e.g., the correlations between two genes) is lacking [12, 13]. We performed GCN analysis (GCNA) to identify delicate topological structure differences between st4 and 4S. GCNA is widely used (e.g. [14]) to detect gene clusters that classify between two states, such as normal and malignant tumors, and to detect responsible genes extracted by comparing two networks. But thus far it has several limitations. First, there is a problem with noise removal and a problem with the removal of low-expression genes in network construction [15]. Usually, genes with low expression are removed (e.g., a cutoff of the bottom 25% of the average expression level, etc. [12, 16–18]). However, during the construction of GCN graphs, blind deletion of genes with low expression may affect the morphology of the graph [15, 19]. Therefore, an appropriate cutoff value should be established [20]. Furthermore, a naive removal of low-degree nodes in a graph may disrupt its functional structure [21]. Second, the information in the GCNA graph is not fully utilized, resulting in an immature topological interpretation. The GCNA approach that has been done so far has been used to identify hubs and gene clusters [22, 23]. Some papers discuss to the detailed connections of genes in network (e.g. [14]), but they concluded to focusing on individual genes or mi-RNAs and did not pay attention to the detection of the topological aspects.

The gene ontology (GO) of each module was also analyzed in previous studies to explore its biological significance; however, when comparing two cancers of the same origin, such as st4 and 4S, the main gene networks were almost identical. In other words, critical differences may reside in node connections (edges) and not in the nodes themselves [22, 24]. To identify such subtle GCN differences, the construction of a GCN graph must be carefully designed.

To construct such a highly accurate and sophisticated GCN graph, as mentioned above, the data must be denoised. We used the resolution of the curse of dimensionality (RECODE) method [25]. In addition, because conventional GCN, which analyzes only adjacent nodes (genes) with the default settings, cannot interpret the topological meaning of the entire graph, we used the generalized topological overlap measure (GTOM) method [26]. The reason for adopting GTOM is that, when the understanding of characteristic subnetworks is important (that may happen in distinguishing 4 and 4S), it is more appropriate to evaluate not only neighboring nodes but also nodes in *k*-th connections ahead. Furthermore, to handle the well-known problem of errors in RNA-sequencing, we employed a specifically designed denoising method of RECODE (not conventional methods by low-expression data cutoff) for the following reasons. One of the parameters in constructing a network from the correlation matrix is the cutoff value for the correlation coefficient, which determines which pair of two TFs is connected. Although noise elimination may not have a large effect on pairs of nodes whose correlations are far from the cutoff value (because nodes with small correlations are cut from the beginning and nodes with large correlations are not cut by a small change), near the cutoff value, the effect by noise elimination seems noticeable and may significantly change the essential part of the network structure. Therefore, we thought it would be more desirable in our analysis to use a method that reduces the data variance such as RECODE, rather than the conventional denoising method that simply cuts off the low-expression data.

Transcription factors (TFs) can influence each other to form a network, but non-TF genes cannot be directly involved in the network [27]. Therefore, it is believed that transcription factors form the core network. It has also been rigorously proven in a mathematical model of gene networks [28] that the network system follows the “core” of the network system. Based on this observation, we analyzed GCN construction using only TFs, which should contribute to the core of the network system.

In summary, we performed GCN analysis using RECODE and GTOM. The biological significance of the obtained subnetworks was vague in the GO analysis, but could be clearly demonstrated by comparison with the adrenocellular single-cell RNAseq analysis of fetal mouse adrena glands and the NB enhancer classification.

Finally, it should be mentioned that the purpose of this study is “to find factors for classifying 4 and 4S as features in the gene network” and “not to propose a new classification method or to compare its classification performance with existing classification methods”. Conventional network analysis of NBL is insufficient for such high-precision graph analysis, and this study is novel in the sense that the subnetworks obtained by topological analysis were meaningful.

## Method

### Preparation

RNA expression analysis, including GCN analysis, was performed based on RNA-sequencing data from 148 primary neuroblastoma tumors sequenced through the TARGET (Therapeutically Applicable Research to Generate Effective Treatments) initiative (phs000467.v1.p1.) and 498 samples from GEO (Gene Expression Omnibus ) as accession ID: GSE49711 [29]. The number of patients was 127 for st4 and 21 for 4S, and the number of genes was 31,849 for TARGET. The number of patients was 183 for st4 and 53 for 4S, and the number of genes was 60,778 for GSE49711. In this study, only 1,531 transcription factors were used based on [30].

The data are downloaded via Genomic Data Commons (GDC) portal site [31], using the Cohort Builder to create a cohort of TARGET-NBL cases (apply filter Project=TARGET-NBL ), then Navigate to the Repository and add the following filters: 15 Workflow Type=STAR - Counts, Access=open. This should result in 162 files for 155 individuals [32]. From this 155, 148 data sets were selected for which clinical data were available. The details of preprocessing are in [33]. In summary, the mRNA Analysis pipeline begins with the Alignment Workflow, which is performed using a two-pass method with STAR. Following alignment, the raw counts files produced by STAR are normalized using three commonly used counts transformations (FPKM, FPKM-UQ, and TPM) along with basic annotations as part of the RNA Expression Workflow. GENCODE v36 was used for gene annotation. The list of downloaded files is in additional file ( 20230518_DWNLD_data_NB_dbGAPfile_list_GDC_NBL ). The data of GSE49711 is downloaded via GEO [34] as a file name of GSE49711_SEQC_NB_MAV_G_log2.20121127.txt.gz. Due to patient privacy issues the raw data was not submitted here. The preprocessing of data was performed via Magic-AceView pipeline, gene expression is measured in sFPKM and transformed to log2 (in details see [29]). Clinical data is also downloaded via GDC portal site, see Data availability.

### Analysis flow

**Fig. 1:**
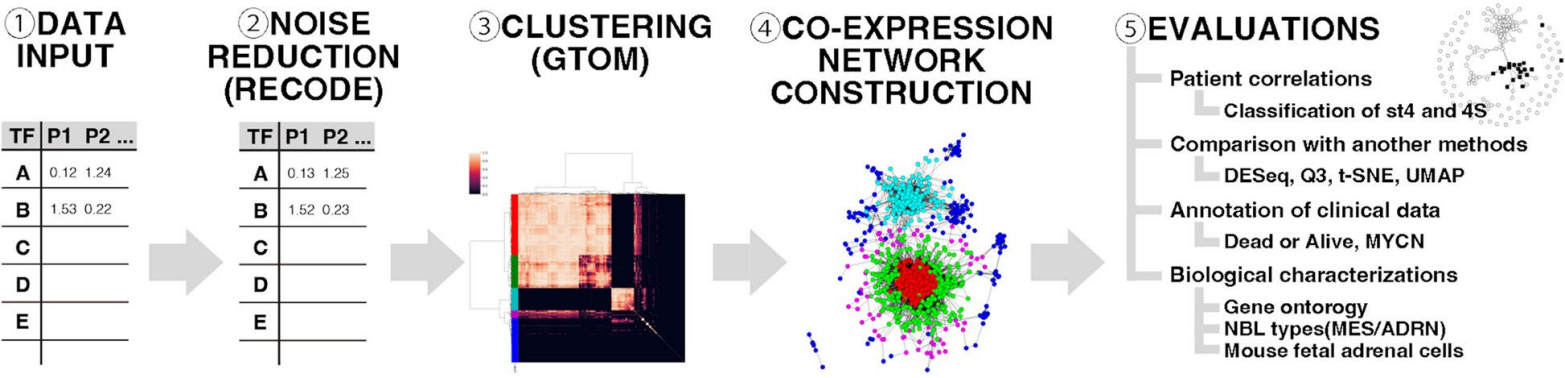
Detailed steps of the study. ① Data input: RNA-sequencing data from 148 primary neuroblastoma tumors. ② Noise reduction: applying RECODE to avoid a curse of dimensionality. ③ Clustering: transformation into an adjacency matrix, calculation of modified matrix by GTOM, and hierarchical clustering by using Ward method. ④ Co-expression network construction: visualization based on the measures by GTOM using python package, igraph. ⑤ Evaluations: several evaluations were performed to discuss obtained clusters

Our analysis flow with RECODE and GTOM is designed as follows (summarized in Fig. 1). (1) RNA-sequencing data from the 148 primary NBL tumors were input. (2) The RECODE method was applied to the RNA-sequencing data to obtain data (whose size is the same as the original data) with noise reduction to avoid the curse of dimensionality. (3-i) An adjacency matrix was computed from the resulting data of step 2 with a given threshold of 0.7. (That is, the (*i*, *j*)-element of the matrix is 1 if the correlation between the *i*-th TF and the *j*-th TF is 0.7 or greater and 0 otherwise.) (3-ii) The GTOM method was applied to the matrix of step 3-i to obtain a matrix (whose size is the same as the original matrix). (3-iii) The average linkage hierarchical clustering was performed on the matrix of step 3-ii using Ward’s method. (4-i) The co-expression network graph was drawn according to the matrix of step 3-i. (That is, the edge between *i*-th node and *j*-th node is drawn if and only if the (*i*, *j*)-element of the matrix is 1.) This was visualized by the Python package igraph. (4-ii) Each node of the graph of step 4-i was colored according to the clusters in step 3-iii. (5) Several evaluations were conducted to discuss the findings in the co-expression networks. A brief description of each method is given below.

### RECODE

RNA sequencing data are high-dimensional with substantial technical noise. This causes a statistical problem known as the curse of dimensionality. In the context of single-cell RNA sequencing (scRNA-seq), Imoto et al. [25] devised a noise reduction method known as the resolution of the curse of dimensionality (RECODE) for high-dimensional data with random sampling noise. It was developed based on high-dimensional statistics. RECODE does not involve dimensional reduction and recovers the expression values for all genes, including genes with low expression. This enables precise delineation of cell fate transitions and identification of rare cells with complete gene information.

We explain the RECODE method more precisely to show its high versatility because we apply RECODE in a slightly different context from [25], that is, in the context of RNA sequencing. Given a matrix date $X=\{x_{ij}\}_{1\le i\le d, 1\le j\le n}\in \mathbb R^{d\times n}$ (in our case, *d* is the number of TFs and *n* is the number of patients), RECODE with a parameter $\ell \in \{1,2,\ldots ,d\}$ defines the modified date matrix $\widetilde{X}_\ell \in \mathbb R^{d\times n}$ by the composition of three operations, namely, the principal component analysis (PCA) coordinate change, a modification/cutoff of eigenvalues with parameter $\ell$, and the inverse PCA coordinate change:

$$\begin{aligned} \widetilde{X}_\ell = \varphi _{X}^{-1}\left(\widetilde{\Lambda }_{X}^{\frac{1}{2}}\Lambda _{X}^{-\frac{1}{2}}\varphi _{X}(X)\right). \end{aligned}$$

Here, $\varphi _{X}$ is the PCA coordinate change given by

$$\begin{aligned} \varphi _X(X)=U_X^T(X-\overline{X}) \end{aligned}$$

with the mean matrix $\overline{X}$ of *X* (i.e. each (*i*, *j*)-element of $\overline{X}$ is the mean $\frac{x_{i1}+\cdots +x_{in}}{n}$ of *i*-th row data of *X*) and the orthogonal matrix $U_X=(u_{X,1},\ldots ,u_{X,d})$ consisting of eigenvectors of the sample covariance matrix $S_X=\frac{1}{n-1}(X-\overline{X})(X-\overline{X})^T$ (i.e. $S_Xu_{X,i}=\lambda _{X,i}u_{X,i}$ with $\lambda _{X,1}\ge \lambda _{X,2}\ge \cdots \ge \lambda _{X,d}$). Hence, $\varphi _X^{-1}(Y)=U_X Y + \overline{X}$. Furthermore, $\Lambda _X$ is a diagonal matrix with diagonal entries by eigenvalues $\lambda _{X,1},\ldots ,\lambda _{X,d}$ and $\widetilde{\Lambda }_X$ is a diagonal matrix with diagonal entries by the modified values $\widetilde{\lambda }_{X,1},\ldots ,\widetilde{\lambda }_{X,d}$ given by

$$\begin{aligned} \widetilde{\lambda }_{X,i}= \left\{ \begin{array}{ll} \lambda _{X,i}-\frac{1}{D-i+1}\sum \limits _{j=i+1}^{D}\lambda _{X,j} \quad & \text {if}\ i\le \ell \\ 0 & \text {if}\ i> \ell \end{array}\right. \end{aligned}$$

with the PCA dimension $D=\min \{n-1,d\}$. There are two types of the curse of dimensionality, and the first one is described as the increasing effect of noise on the data variance in the limit $d\rightarrow \infty$. RECODE regards the first $\ell$ principal components (PCs) as the essential part and the other PCs as the noise part. In [25], it is proven that there is an $\ell$ to minimize the effect of noise on the data variance. We also employed the optimal $\ell$ in our computation. The second type of the curse of dimensionality is seen in the non-convergence of $\lambda _{X,i}$ with noise to the corresponding eigenvalue without noise in the limit $d\rightarrow \infty$ in the case $d\gg n$, which the classical PCA also suffers from. However, it is proven in [25] that the modified value $\widetilde{\lambda }_{X,i}$ with noise converges to the eigenvalue without noise.

### GTOM

Network methods are useful for representing the interactions of genes. Yip and Horvath [26] proposed GTOM(*k*) (generalized topological overlapping measure of order $k+1$), which is a clustering method that uses not only the first-order connections (i.e., the adjacent connections between 2 nodes), but also a measure of topological overlap based on $(k+1)$-th order neighborhoods. More precisely, the matrix $T=\{t_{ij}^{(k)}\}_{1\le i,j\le d}$ obtained by GTOM(*k*) from an adjecency matrix $A=\{a_{ij}\}_{1\le i,j\le d}$ is determined by the formula

$$\begin{aligned} t_{ij}^{(k)}= \left\{ \begin{array}{ll} \frac{\vert N_{k+1}(i)\cap N_{k+1}(j)\vert +a_{ij}}{\min \{\vert N_{k+1}(i)\vert , \vert N_{k+1}(j)\vert \} +1-a_{ij}} \quad & \text {if}\ i\ne j\\ 1 & \text {if}\ i=j, \end{array}\right. \end{aligned}$$

where $N_{k+1}(i)$ is the set of nodes (excluding *i* itself) that are reachable from *i* within a path of length $k+1$ and $\vert A \vert$ is the cardinality of a set *A*. Notice that even in the case $a_{ij}=0$, if the number of paths of length $k+1$ from the *i*-th node to the *j*-th node is large, then $t_{ij}^{(k)}$ can be close to 1. To quantify the separation ability of GTOM(*k*) in a given decomponsition $\{1,2,\ldots ,d\}=\mathcal A\cup \mathcal B$ of all nodes by disjoint groups $\mathcal A$ and $\mathcal B$, we also consider the measure of mean difference $\textrm{GTOMdiff}_k (\mathcal A,\mathcal B)$ of order $(k+1)$ (with respect to $\mathcal A$, $\mathcal B$) given by

$$\begin{aligned} \textrm{GTOMdiff}_k(\mathcal A,\mathcal B)=\textrm{GTOMscore}_k(\mathcal A,\mathcal A)-\textrm{GTOMscore}_k(\mathcal A,\mathcal B), \end{aligned}$$

where $\textrm{GTOMscore}_k (\mathcal C,\mathcal D)$ for groups $\mathcal C$, $\mathcal D$ in $\{1,2,\ldots ,d\}$ is the measure of interconnectedness of $\mathcal C$ and $\mathcal D$ given by

$$\begin{aligned} \textrm{GTOMscore}_k (\mathcal C,\mathcal D) =\frac{\sum _{(i,j)\in \mathcal C\times \mathcal D, \; i\ne j}t_{ij}^{(k)}}{\vert \{ (i,j)\in \mathcal C\times \mathcal D : i\ne j\}\vert } . \end{aligned}$$

Since high values of $\textrm{GTOMdiff}_k$ indicate a good separation between the two groups $\mathcal A$ and $\mathcal B$, we employ *k* as the value that maximizes $\textrm{GTOMdiff}_k$.

### Gene Ontology (GO) and cell signature analyses

The PANTHER Overrepresentation Test (Released 20230705) [35–37] was used for ontology-based biological process analyses of TF clusters. Gene signature analysis of each cluster was performed in two ways according to previous studies. One was a comparison with two types of NBLs classified by superenhancers, and the other was a comparison with single-cell RNAseq of fetal mouse adrenal cells. Van Groningen et al. showed that Neuroblastoma is composed of two super-enhancer-associated differentiation states [38]. Furthermore, super-enhancer-associated TF networks underlie lineage identity. NBLs are epigenetically classified as adrenergic (ADRN) and mesenchymal (MES) and each is regulated by independent superenhancers.

Hanemaaijer et al. conducted a single-cell RNAseq analysis of fetal mouse adrenal glands at each developmental stage and the genes that characterized each cell were identified [39] (see Additional file 1). They classified fetal mouse adrenal cells into five groups: cortex, medulla, endothelium, stroma, and immune, and further divided them into subgroups according to their differentiation stage. We counted and tabulated which of these signatures the transcription factors in our cluster corresponded to.

## Results

### Gene correlations for clustering

**Fig. 2:**
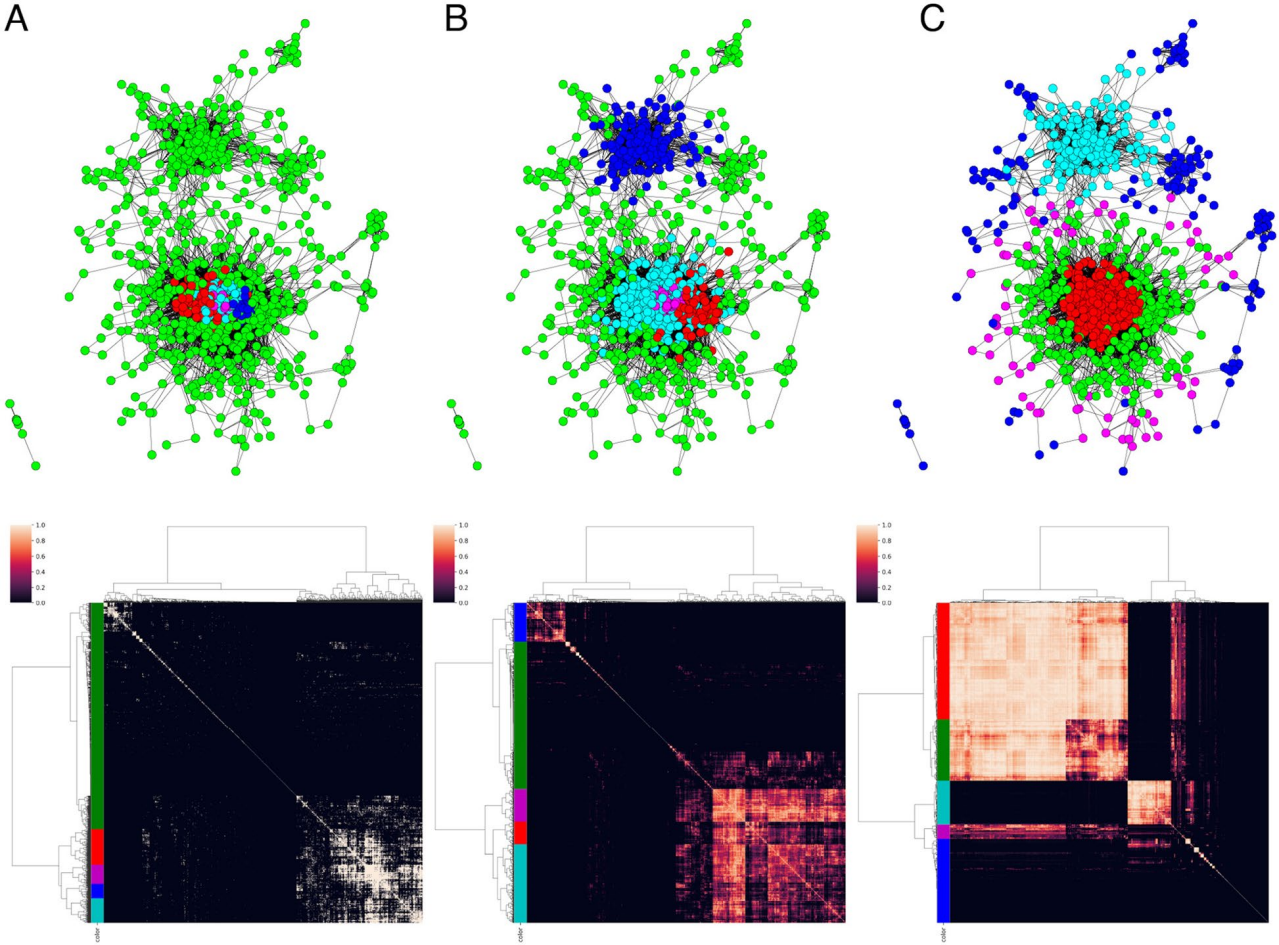
The upper figure displays the matrix as a network graph with RECODE, in which the nodes consist of 1,531 TFs and each edge between the nodes is drawn if the correlation between the corresponding TFs is 0.7 or greater, where TFs that have fewer than five connections with other TFs are not displayed. The color of each node is determined by hierarchical clustering of the matrix, which is calculated as A: GTOM(0), B: GTOM(1), and C: GTOM(2). The lower figure displays the clustering heatmap of each GTOM

We first calculated the Pearson correlation coefficient between each pair of 1,531 TFs in the downloaded data, and created a $1531\times 1531$ correlation matrix. Figure 2 displays the correlation matrix as a TF-GCN graph, in which the nodes consist of 1,531 TFs and each edge between the nodes is drawn if the correlation between the corresponding TFs is 0.7 or greater. The upper figure in Fig. 2 displays the matrix as a network graph. In the graph, TFs that have fewer than five connections with other TFs are not displayed. The lower figure in Fig. 2A is an adjacency matrix of the TFs. Each entry is 1 if the corresponding correlation coefficient is 0.7 or greater and 0 otherwise. Note that hierarchical clustering is performed for each matrix using the Ward method.

**Fig. 3:**
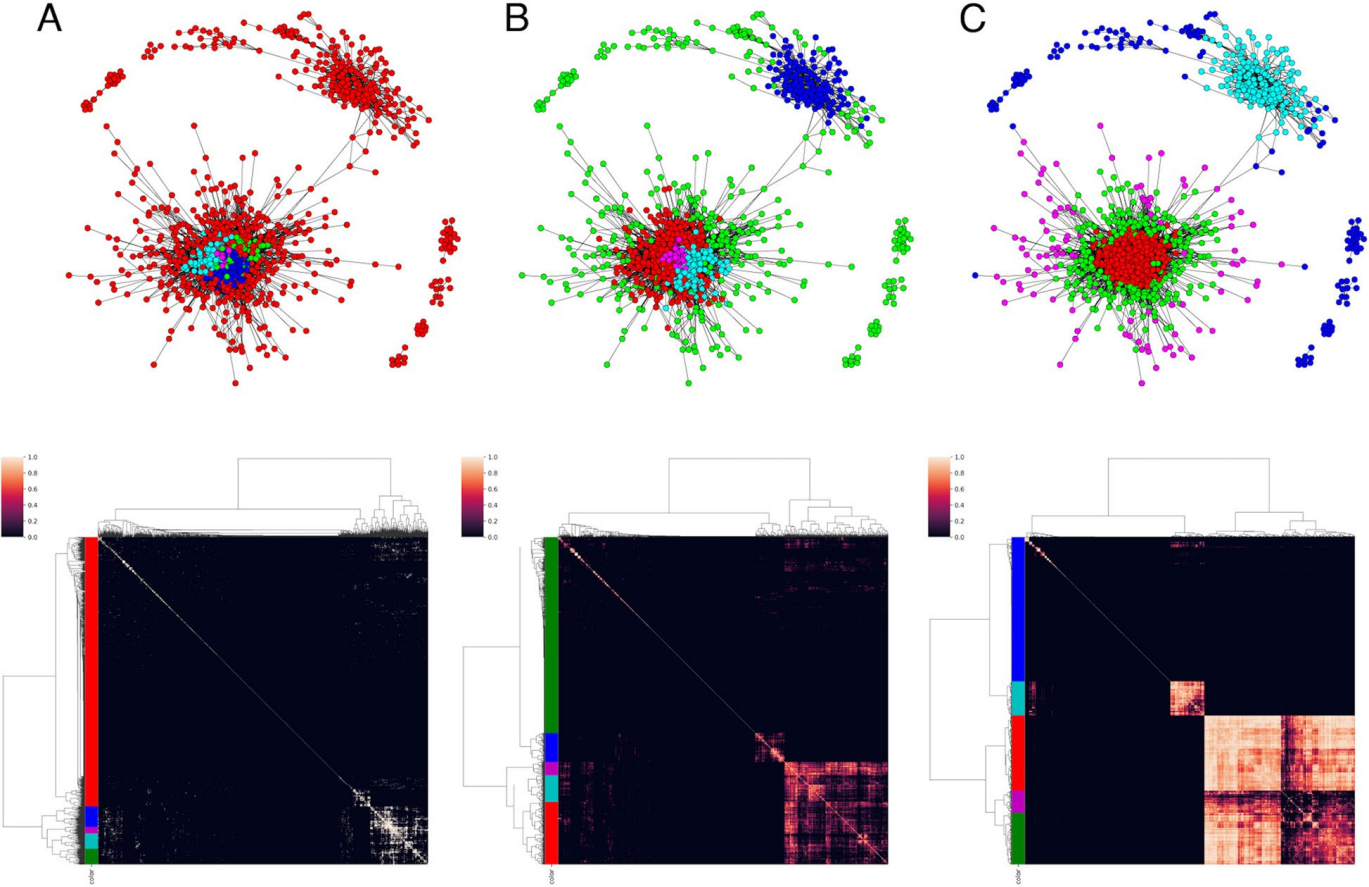
The upper figure displays the matrix as a network graph without RECODE, in which the nodes consist of 1,531 TFs and each edge between the nodes is drawn if the correlation between the corresponding TFs is 0.7 or greater, where TFs that have fewer than five connections with other TFs are not displayed. The color of each node is determined by hierarchical clustering of the matrix, which is calculated as A: GTOM(0), B: GTOM(1), and C: GTOM(2). The lower figure displays the clustering heatmap of each GTOM

The TF-GCN graph was used to analyze the graph structure. In this case, the GCN was reconstructed by GTOM analysis (GTOM-GCN) to focus on the robust topological structure of the graph. In Fig. 2, the matrix calculated by GTOM(0) (the adjacency matrix) and its cluster analysis results are shown in A, and these objects calculated by GTOM(1) and GTOM(2) are drawn in B and C, respectively. Furthermore, we performed a hierarchical cluster analysis for GTOM-GCN via the Ward method and divided the TFs into five major clusters (colored red, green, cyan, magenta, and blue). The number of clusters used in this analysis was five, as several indices (e.g. the silhouette coefficient) were used as references, and a characteristic subnetwork was found in the network graph using GTOM(2). The number of TFs in each cluster is listed in Table 2.

The red cluster was strongly related to the nodes within it. The cyan cluster is the same. The green and magenta clusters were related to the red and cyan clusters, but they exhibited a weak intercorrelation within their own clusters. In the graphical display, the red cluster is surrounded by green and magenta clusters. The blue cluster was considered to be a collection of TFs that were not correlated with other TFs. Table 2 also shows that the blue cluster contains more TFs with low expression levels than the other clusters.

The reasons for giving a cutoff value of 0.7, a number of clusters of 5, and GTOM(2) as the basis for this study are described below. As shown in the figure (see Additional file 7), the number of clusters 2 or 3 is appropriate by commonly used indices such as silhouette coefficient, but since the purpose of this experiment is to extract characteristic subclusters, the number of clusters is set to 4 or 5 in order to divide the data into a larger number of clusters to the extent that it is meaningful. Then, the GTOMscore was calculated for cutoff values in range [0.5, 0.9] and $k=0,1,2,3$, to quantitatively confirm the relationship between the clusters. For example, the Table 1 shows the GTOMscores for the cutoff value of 0.7, the number of clusters of 5, and GTOM(2).

**Table 1:** GTOMscores for cutoff value of 0.7, the number of clusters of 5, and GTOM(2)

	Red	Green	Magenta	Cyan	Blue
Red	0.940	0.897	0.408	0.006	0.005
Green		0.586	0.182	0.003	0.003
Magenta			0.0639	0.0367	0.005
Cyan				0.809	0.036
Blue					0.014

The diagonal component is the connectivity within each cluster, showing that red and cyan are strongly connected. The green cluster is moderately connected to itself but is strongly connected to red, magenta is reasonably strongly connected to red, and blue is weakly connected to both clusters. We calculated the GTOMscore for several parameters in order to find a network with a reasonably distributed number of nodes in each cluster, and we find a cutoff value and an index *k* as the one that well reflects the following three characteristics:

- More than two distinct core networks (e.g. red, cyan)
- Subnetworks contributing to the core network (e.g. green, magenta)
- Networks with some other characteristics (e.g. blue)

**Fig. 4:**
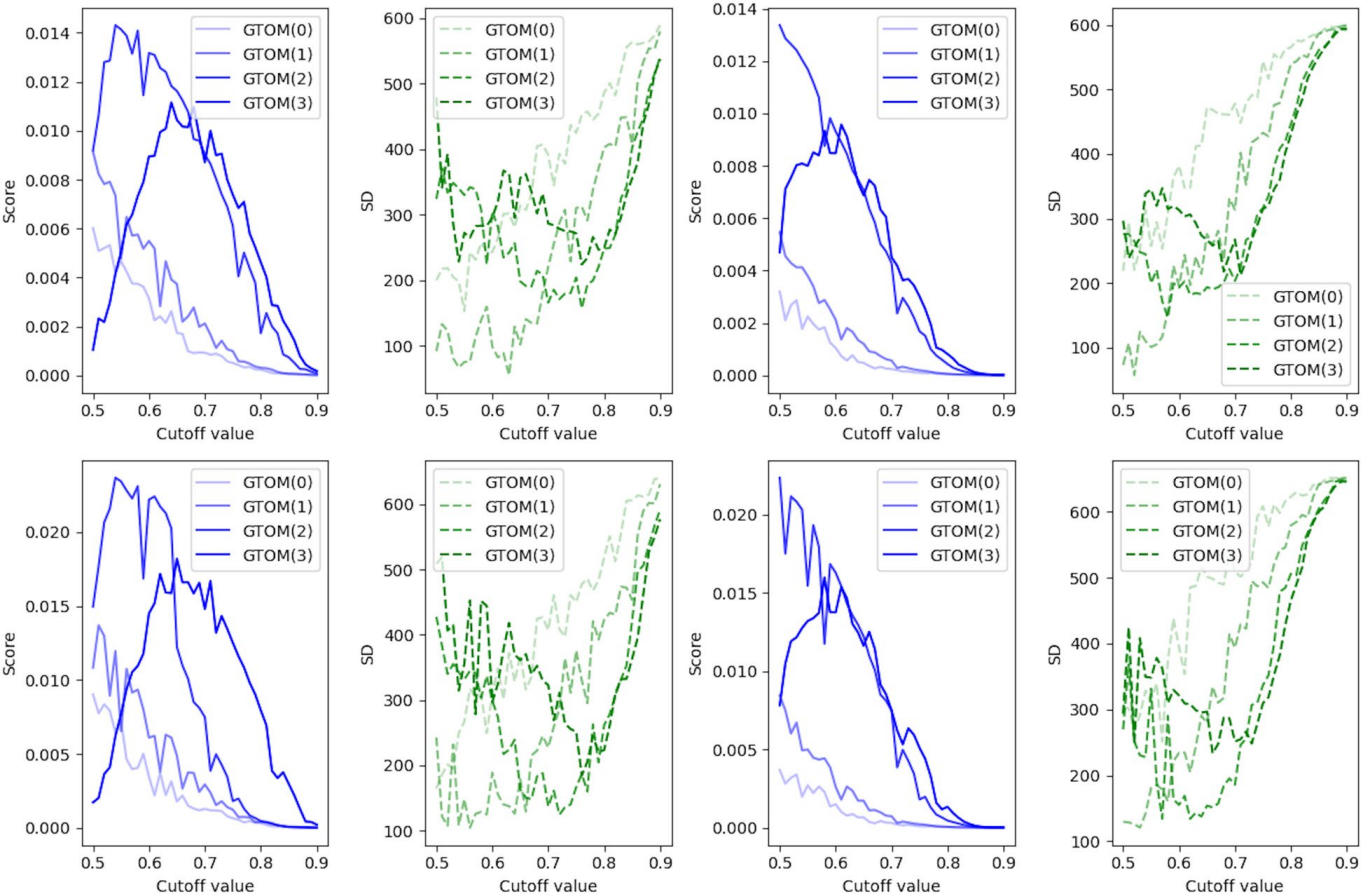
The total score of GTOMscore (blue line) and the standard deviation of the number of nodes in each cluster (green line) for the case with RECODE and 5 clusters (upper left), with noRECODE and 5 clusters (upper right), with RECODE and 4 clusters (lower left), with noRECODE and 4 clusters (lower right)

To find appropriate parameters, we further (introduce and) calculate the total score of GTOMscores defined by

$$\begin{aligned} S_k = \frac{1}{d_C}\sum _{(i,j)\in C^2, \, i<j}r_i r_j \big \{G_k(i,i) G_k(j,j) (1-G_k(i,j)) +G_k(i,i) (1- G_k(j,j)) G_k(i,j)\big \}, \end{aligned}$$

where *C* is the numbered set of all clusters obtained by the clustering, $d_C$ is the total number of pairs of different clusters (i.e. $\vert \{(i,j)\in C^2: i<j\}\vert$), $G_k(i,j)=\textrm{GTOMscore}_k(\mathcal A_i,\mathcal A_j)$ (denoting by $\mathcal A_i$ the set of all nodes in the *i*-th cluster), and $r_i$ is the ratio of the number of nodes in the *i*-th cluster (i.e. $\frac{\vert \mathcal A_i\vert }{\sum _{j}\vert \mathcal A_j\vert }$). By this equation, we can find the characteristic clusters. More precisely, the first term $G_k(i,i) G_k(j,j) (1-G_k(i,j))$ becomes higher if the two clusters *i* and *j* are different core networks, and the second term $G_k(i,i) (1- G_k(j,j)) G_k(i,j)$ becomes higher if some subnetwork has a weak connection to itself, but strongly connected to *i*. The upper left graph in Fig. 4 shows the score $S_k$ for the data with RECODE, 5 cluster and the cutoff values from 0.5 to 0.9 and $k=0,1,2,3$, displaying the case $k=2$ gives higher scores. Moreover, we consider the standard deviation (SD) of the number of nodes in each cluster because even if the score $S_k$ is high, if almost all nodes are included in one cluster, then we cannot detect a characteristic subnetwork. Figure 4 also shows the SD for each case, which displays that the SDs in the range of cutoff value [0.70, 0.76] are relatively low. Combining the score $S_k$ and the SD, we determined the cutoff value 0.7 and $k=2$ that realize the higher total score and the lower SD.

Finally, to confirm the effect of RECODE in Fig. 2, network graphs and clustering heatmaps obtained by GTOM without RECODE are illustrated in Fig. 3, where the colors are used for their respective clustering and have no relationship to Fig. 2. Moreover, total scores and SDs are summarized in Fig. 4.

### Patient correlations

To determine whether each TF cluster contributes to the difference between st4 and 4S, we created an intersample (Spearman) correlation graph using the TFs from each cluster. We used a Spearman correlation rather than a Pearson correlation because gene expression levels generally follow a power-law distribution [40].

**Fig. 5:**
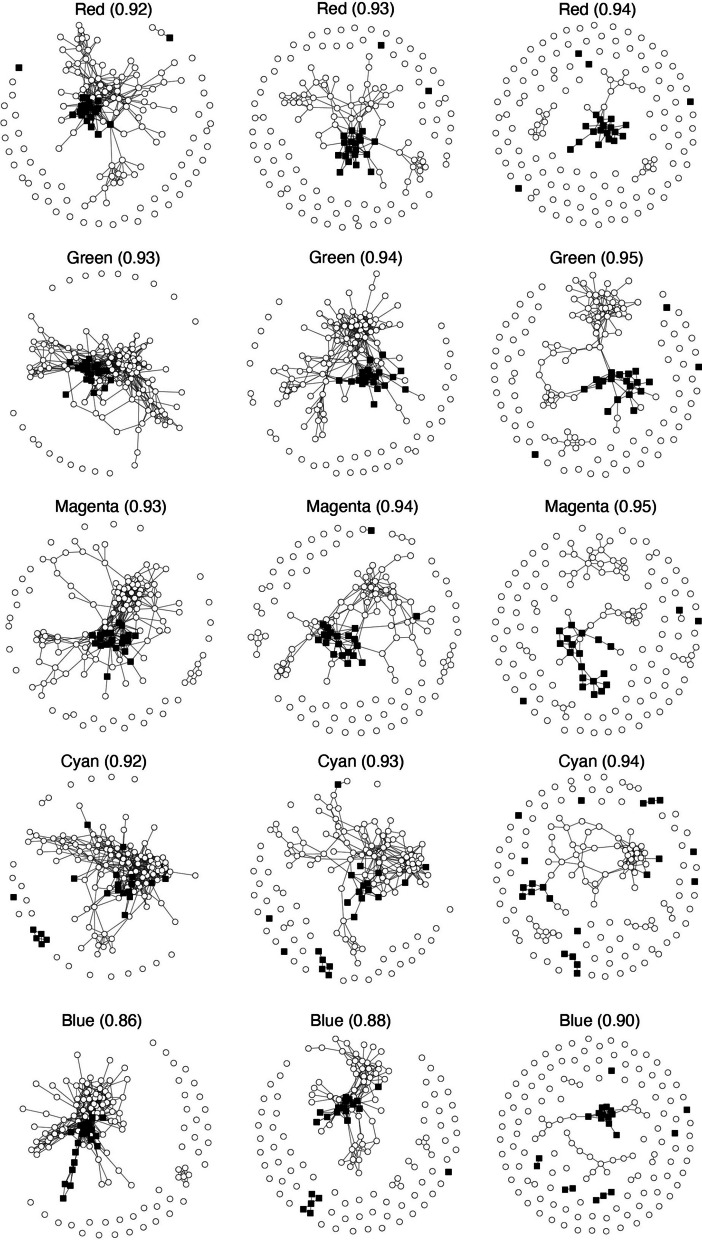
The visualization of patient correlations as a network graph by using five clusters. Each node corresponds to a patient of $\circ$ for Stage 4 and $\blacksquare$ for Stage 4S, and it connects each pair of patients with a Spearman correlation greater than the value in parentheses

Figure 5 presents a visualization of patient correlations as a network graph. No separation was observed in the cyan and blue clusters; however, the green cluster successfully separated st4 and 4S. The red and magenta clusters also separated st4 and 4S.

**Fig. 6:**
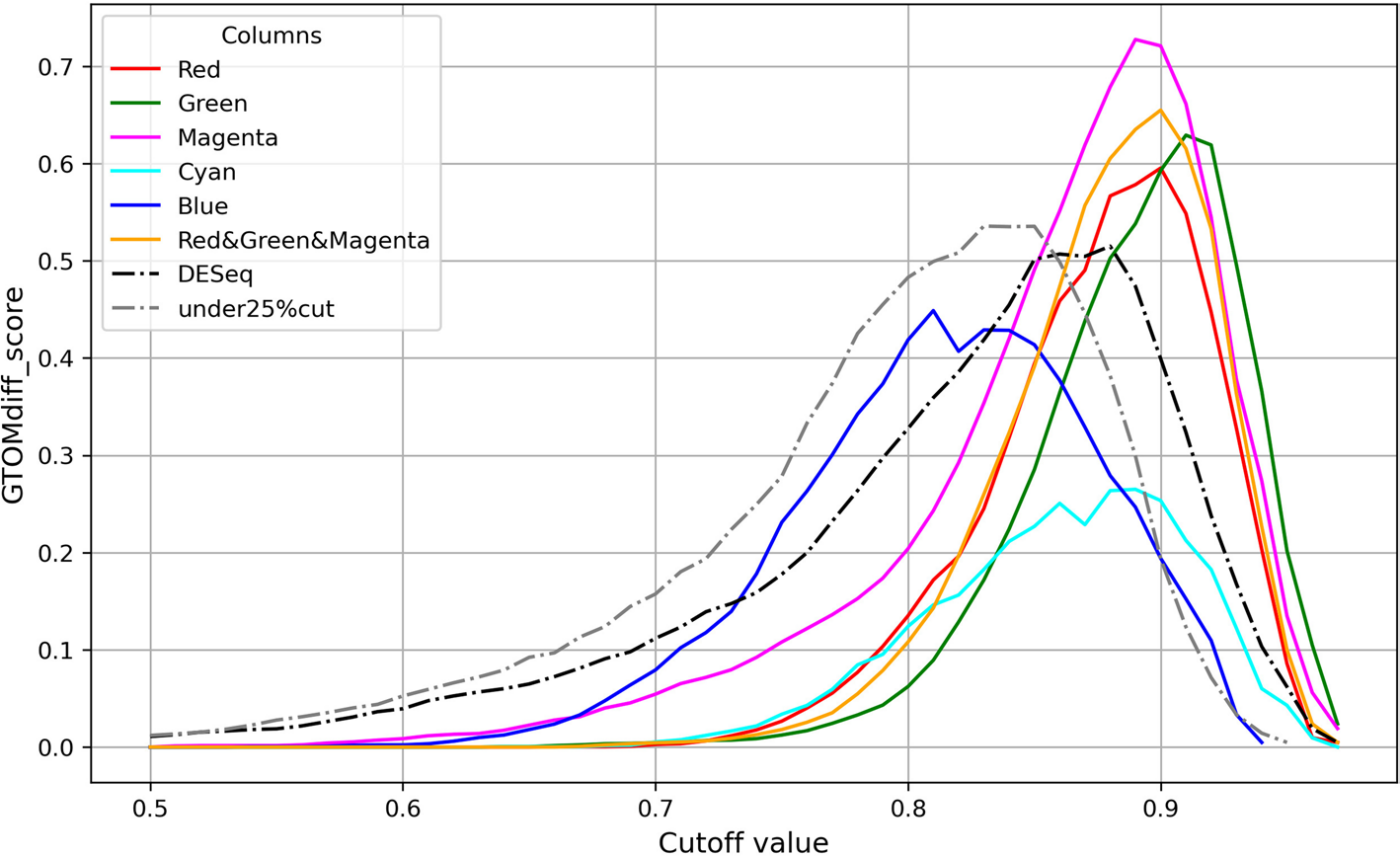
The graph of the difference of GTOM score between group st4 and 4S for the patient correlation network created for each cluster depending on the cutoff value. The two dotted lines represent the GTOMscore between groups st4 and 4S for the patient correlation network created by DESeq and the third quantile (Q3)

As quantitative evaluations, we calculate a difference GTOM score of group st4 and 4S, $\textrm{GTOMdiff}_0(4,4S)$, for each cut-off value of correlations from 0.50 to 0.95. Note that although we call it the GTOMscore, GTOM is not relevant since this score is calculated using the adjacency matrix (i.e. GTOM(0)). Figure 6 displays the scores, and we can find that the clusters red, green and magenta give a well-separation of st4 and 4S.

### Comparison with other methods

**Fig. 7:**
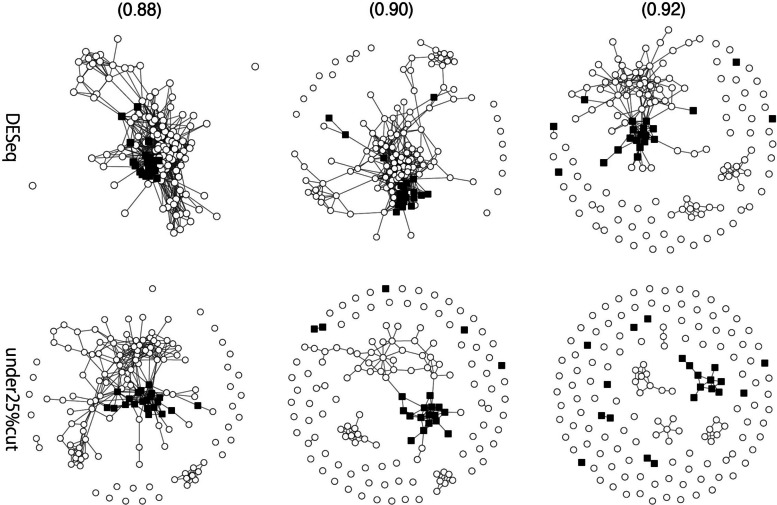
Upper network graphs are from patient Spearman correlations of TFs for which the difference in expression between st4 and 4S was significant (adjusted $p<0.01$) according to DESeq. Lower network graphs are from patient Spearman correlations of TFs in the third quartile (Q3) of gene expression levels. Each node corresponds to a patient of $\circ$ for Stage 4 and $\blacksquare$ for Stage 4S, and it connects each pair of patients with a Spearman correlation greater than the value in parentheses

To show that green and red clusters strongly influence the difference between 4 and 4S, we performed two control experiments and two well-used clustering methods. First, we calculated patient correlations with 239 TFs for which the difference in expression between st4 and 4S was significant (adjusted $p<0.01$) via the R package “DESeq2” (Fig. 7). Second, patient correlations were calculated for 1,149 TFs in the third quantile (Q3) of gene expression levels (i.e., the TFs representing the bottom 25% of the median gene expression levels were removed) (Fig. 7). Table 2 lists the number of TFs of interest in each cluster. The GTOM scores between group st4 and 4S for the patient correlation network created by DESeq and Q3 are also illustrated in Fig. 6.

Moreover, for reference as a comparison with other clustering methods, tSNE [41] and UMAP [42] which display the projection onto a two-dimensional plane were applied to the same data with RECODE, and the results are shown in Additional file 8 with the same colors as GTOM(2) with RECODE. It should be mentioned, however, that the choice of parameters is an important issue for these methods, but the parameters used in these experiments are the default ones in Python.

**Table 2:** Breakdown of the number of TFs in clusters classified by GTOM(2) and cutoff value 0.7, and the number of TFs in each cluster used in the control experiment (DESeq and third quartile (Q3))

	$\#$	DESeq2	Q3
Red	556	65 (11.7$\%$)	543 (97.7$\%$)
Green	298	64 (21.5$\%$)	259 (86.9$\%$)
Magenta	68	11 (16.2$\%$)	57 (83.8$\%$)
Cyan	209	38 (18.2$\%$)	142 (67.9$\%$)
Blue	400	61 (15.3$\%$)	148 (37.0$\%$)
total	1531	239 (15.6$\%$)	1149 (75.0$\%$)

### Annotation of clinical data on patient correlation networks

**Fig. 8:**
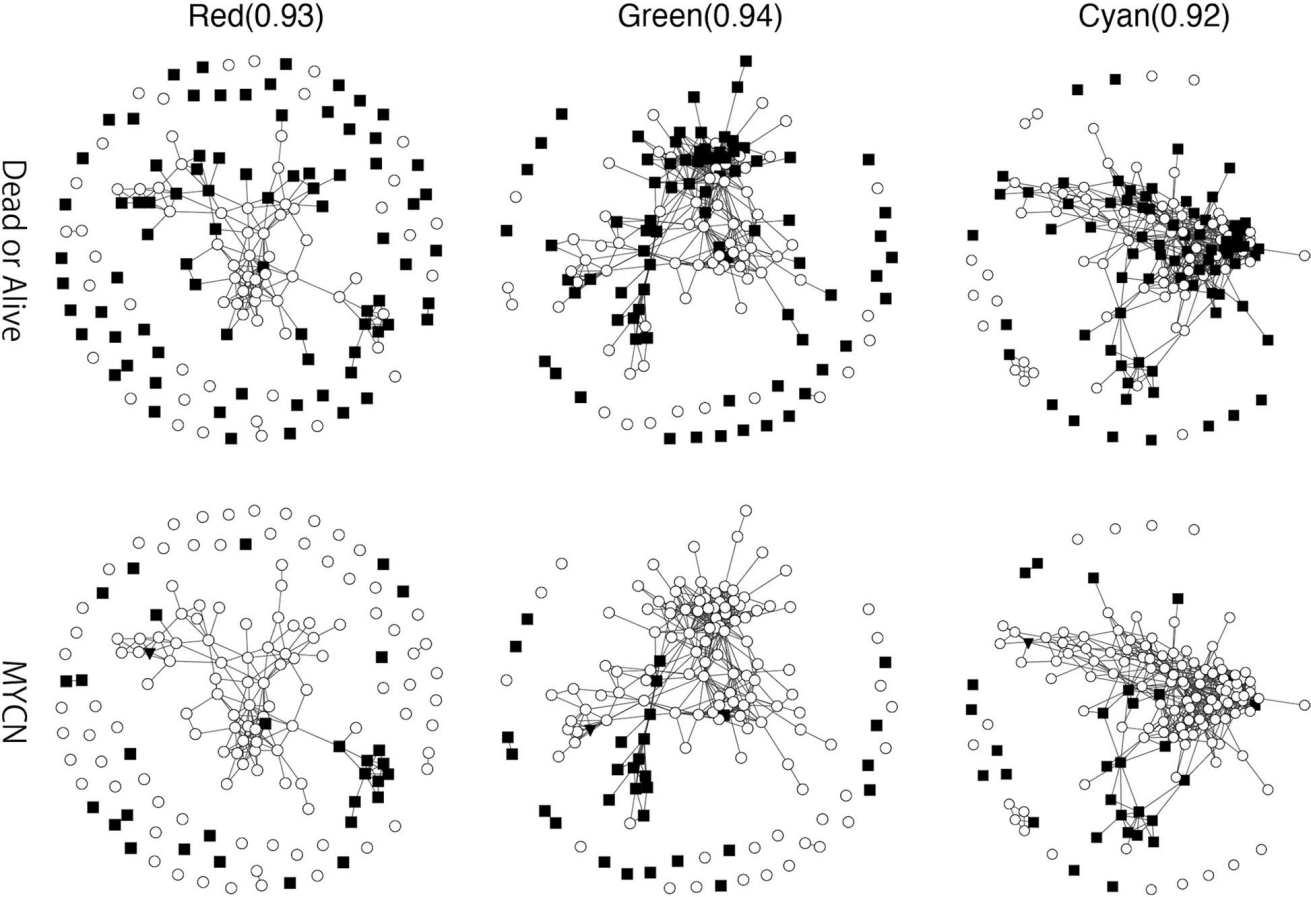
Each node in the upper network graphs corresponds to a patient, whose vital prognosis state is $\circ$ for Alive and $\blacksquare$ for Dead. Each node in the lower network graphs corresponds to a patient whose MYCN state is indicated by $\circ$ for not amplify, $\blacksquare$ for amplify, and $\blacktriangledown$ for unknown. It connects each pair of patients with a Spearman correlation greater than the value in parentheses

Next, we superimposed the following clinical data on the patient correlation graphs obtained above: prognosis vital state (dead/alive), diagnostic category (neuroblastoma, ganglioneuroblastoma nodular, ganglioneuroblastoma intermixed, unknown), MYCN amplification, and mitotic-Karyorrhectic Index (MKI). In Fig. 8, we examined which of these indicators was reflected in the graphs. In the classification by MYCN amplification status, some patients with amplification appeared as a subcluster in the green and red TF clusters but not in cyan. The vital state, the diagnostic category, and MKI did not show any characteristic clustering.

### Biological characterization of 5 clusters

We performed a GO analysis of each cluster through an overrepresentation test using the PANTHER web app by submitting gene IDs (see Table 3 and Additional files 2 for more detail). All 1,531 TFs were used as reference genes. The green and magenta clusters did not have GOs with an FDR (false discovery rate) less than 0.05 and a clear biological function. In the blue cluster, the top GO terms ranked by fold enrichment values were related to brain, nerve, and limb development. In the cyan cluster, the top GO terms were related to blood, immunity, blood vessels, and cardiac development. In the red cluster, all GO terms with an FDR $<0.05$ were underrepresented. This may have occurred because of the large number of genes in the red cluster ($N=556$), which prevents the evaluation of GO characteristics compared with those of the entire group of TFs.

**Table 3:** Characteristics of the GO terms in each cluster. See Additional file 2 for the detail of the list of GO

	GO biological process
Red	under-representation
Green	no significant GO
Magenta	no significant GO
Cyan	pigmentation, fat cell, B cell, insulin receptor signal, smooth muscle
Blue	neuron, kidney development, helper-T

Since GO did not adequately characterize the clusters, we made the following two comparisons with previous studies. One was to evaluate the similarity to the two types of neuroblastoma (MES/ADRN) and the other was to evaluate the similarity to the 5 types of fetal mouse adrenal cells.

The two epigenetic classifications of neuroblastoma cells include the MES and ADRN types, each of which is regulated by an independent superenhancer. The MES type is presumed to have differentiated from the Schwan cell precursor (SCP), while the ADRN type is presumed to have differentiated from sympathetic neurons [38, 43]. MES-type signatures were mostly found in the cyan cluster, followed by the blue cluster, but were rarely found in the red, green, and magenta clusters. ADRN-type signatures belonged mostly to the red and green clusters and were also found in the blue cluster, but were rarely found in the cyan cluster (see Table 4 and Additional file 3).

Compared with the gene signatures of mouse fetal adrenal cells [39], the most abundant cluster of genes belonged to the medulla, consistent with the fact that NBL is a malignant tumor of adrenal medullary origin. Next, those belonging to the endothelial, stromal, and immune were found with equal amounts, but only a few were included in the cortex. While medulla signatures were found equally in almost all clusters (except magenta), endothelial, stromal, and immune signatures were mostly found in the cyan cluster (see Table 4 and Additional file 4).

**Table 4:** Breakdown of TFs classified by NBL types and Mouse fetal adrenal cells belonging to each cluster

	NBL types		Mouse fetal adrenal cells				
	MES	ADRN	cortex	medulla	endothel	stroma	immune
Red	1	10	4	22	5	5	1
Green	2	13	6	18	12	7	6
Magenta	1	3	2	8	3	2	0
Cyan	28	2	9	21	28	24	30
Blue	8	10	9	24	5	22	17
Total	40	38	30	93	53	60	54

### Placement of HOX genes in GCN graphs

Since genes in intermediate networks such as the green cluster contain information that distinguishes st4 from 4S, we examined what genes are contained in the green, magenta, and blue clusters that are off-center of the network. We found that this intermediate network contains a large number of HOX genes, and we explored them on our graph.

HOX genes are marked on the TARGET-NBL co-expressed gene graph (see Additional file 9A). The HOXA and B groups are at the edges of the cyan cluster (MES-type NBL signature); the B group is also inside the cyan cluster, while the A group forms a slight distant from it. Group D differs from these in that it is located on the edges of the cyan cluster, but also on the edges of the red cluster (ADRN-type NBL signature). The HOXC group differs from these in that it clusters only in the vicinity of the red cluster. To see the effect of RECODE, we also created a graph without RECODE and examined the placement of HOX genes (Additional file 9B). The graph without RECODE appears to be more skewed with overall distortion. The arrangement of the HOXD group is more clustered in a part of the graph than with RECODE, and is not interspersed among the cyan and red clusters.

To see the reproducibility of the graph structure, the HOX genes were also marked on a graph generated with another set of data, GSE49711. The HOXA and B groups are clustered around the cyan cluster, which is consistent with the TARGET data, while in GSE49711, the placement of the HOXD group on the graph differs from the TARGET data, creating distant clusters with no connection between the red and cyan clusters (Additional file 10).

### Reproducibility

**Fig. 9:**
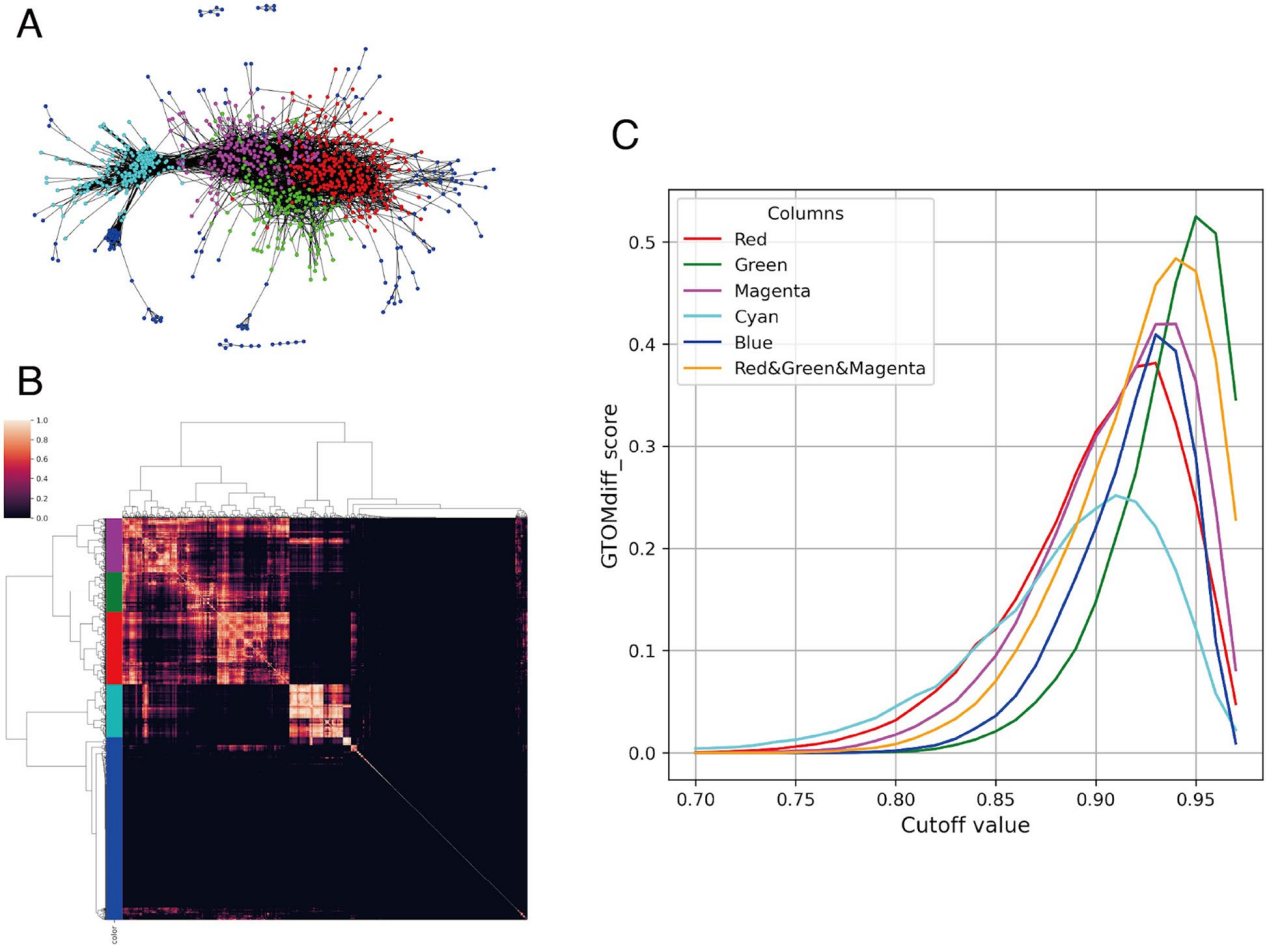
**A** The network graph created by TPM transformed GSE49711 data, in which the nodes consist of 1,531 TFs and each edge between the nodes is drawn if the correlation between the corresponding TFs is 0.65 or greater, where TFs that have fewer than five connections with other TFs are not displayed. The color of each node is determined by hierarchical clustering of the matrix, which is calculated as GTOM(2). **B** The clustering heatmap of GTOM(2). **C** The graph of difference of GTOMscore between group st4 and 4S for the patient correlation network created for each cluster depending on the cutoff value

To demonstrate the reproducibility of the results, we performed the same experiment on GSE49711, which is a different set of data from TARGET. Note that the original data obtained for TARGET was TPM, while GSE49711 was $\log _2(1+\textrm{FPKM})$. It would be appropriate to perform an inverse log transformation to convert FPKM to TPM before applying our method, but we cannot ignore the effect of round errors added by these transformations. In fact, if we apply RECODE after converting to TPM and perform a cluster analysis using GTOM(2) with a cutoff value of 0.65, we obtain the results in Fig. 9 and Table 5.

**Table 5:** Breakdown of the number of TFs in each cluster resulting from cluster analysis by TARGET and GSE49711. The rate in parentheses is the ratio of the number of nodes in each cluster in GES49711 to the number of nodes in each cluster in TARGET

TARGET$\backslash$GSE	Red	Green	Magenta	Cyan	Blue	Total
Red	218 (39.2%)	127 (22.8%)	104 (18.7%)	3 (0.5%)	104 (18.7%)	556
Green	56 (18.8%)	17 (5.7%)	61 (20.5%)	4 (1.3%)	160 (53.7%)	298
Magenta	0 (0.0%)	1 (1.5%)	18 (26.5%)	4 (5.9%)	45 (66.2%)	68
Cyan	0 (0.0%)	3 (1.4%)	8 (3.8%)	145 (69.4%)	53 (25.4%)	209
Blue	0 (0.0%)	4 (1.0%)	15 (3.8%)	46 (11.5%)	335 (83.8%)	400
Total	274	152	206	202	697	1531

The Fig. 9 shows that, despite a cutoff value of 0.65 (lower than 0.7), many TFs belong to the blue cluster, which has few connections. Furthermore, the network structure around red, such as the green and magenta clusters observed in TARGET, was not reproduced. On the other hand, the combined clusters (red, green and magenta) and cyan clusters showed reproducibility of about $65.3\%$ and $69.4\%$, respectively. By the Figure 9-C, the GTOMdiff, which distinguishes between st4 and 4S, also reproduced that the combined cluster gives better accuracy.

## Discussion

We analyzed NBL using highly accurate and designed GCN graphs created by RECODE and GTOM, which consisted of only TFs. By comparing st4 and 4S, we attempted to clarify what causes the spontaneous regression of NBL.

When a characteristic subnetwork is extracted from a network consisting of an adjacency matrix of TFs, well-characterized clustering cannot be achieved using information only from adjacency (1-step neighboring nodes) (Fig. 2A). Figure 2C shows that the GTOM (multistep neighboring nodes) was valid. In fact, five characteristic subnetworks were identified after the calculation of the overlapping measure from the information of the nodes connected within 3 steps (i.e., GTOM(2)), and clustering of the matrix was created from the overlapping measure.

The red and cyan clusters exhibited strong internal connections; the green clusters had weak internal connections but strong connections with the red; the magenta cluster had even weaker connections and was located around the red and green, whereas the blue clusters showed weak overall connections. The green cluster surrounded the red cluster, and the magenta cluster further surrounded it. Another feature was that the cyan cluster had few connections with the red, green, and magenta clusters.

Figure 5 shows that the red or green cluster clearly indicates TF separation between st4 and 4S. Notably, 4S patients were strongly correlated (0.95 for the green cluster and 0.94 for the red cluster). On the other hand, there was no clear separation by the cyan cluster, indicating that the transcription factors contributing to the separation of st4 and 4S are contained in the red and green clusters.

Table 2 shows that TFs with significant differences in DESeq (i.e., those with large differences in expression between st4 and 4S) did not belong biasedly to a specific cluster. Moreover, Fig. 7 shows that in the patient correlation graph with such TFs, there is no clear connected subgraph when the Spearman correlation exceeds 0.92 (which is less than the critical cutoff value of 0.94 for the red cluster and 0.95 for the green cluster). Furthermore, Fig. 8 also shows that in the patient correlation graph of patients whose gene expression cuts off the bottom 25%, no clearly connected subgraph was observed when the Spearman correlation was greater than 0.90.

In the construction of a network graph, it is indispensable to consider the effect of noise. Figure 3 shows the results of GTOM without applying RECODE. In this case, under the same cutoff value of 0.7, many TFs were included in the blue cluster, a gathering of less linked nodes to major clusters, in GTOM(2) (Fig. 3C). This may even remove the TFs contributing to the separation of st4 and 4S, so careful handling of noise is essential. RECODE overcomes the curse of dimensionality by performing singular value decomposition, slightly reducing the portion with large singular values and setting the portion with small singular values to 0 (considered noise), and restoring the original data from there. This suppresses the increase in variability due to noise with respect to the original data and produces the effect of increasing correlations between TFs. This approach would allow correlations within the same population to enhance each other, thereby enabling more accurate separation of populations with the same characteristics.

The biological significance of the 5 clusters found in the NBL TF network by GTOM with RECODE was analyzed. No specific terms were found in the blue, green, or red clusters in the GO analysis. Additionally, no terms were found that directly represented the adrenal medulla. In the gene signature that classifies NBLs into two types, ADRN and MES [38], ADRN signatures were predominantly in the red and green clusters, while MES were predominantly in the cyan cluster, showing mutually exclusive trends. The two major subnetworks in our graph (one is the union of red, green and magenta, the other is cyan) were thought to represent the ADRN and MES type superenhancer-regulated TF systems, respectively. Compared with fetal mouse adrenal cells [39], the TFs in our cluster had predominantly medulla signatures. The magenta and red clusters had almost exclusively medulla signatures; the green cluster contained medulla and endothelial signatures; and the cyan cluster had each signature except for the cortex. According to these comparisons, the red, magenta, and green clusters are associated with the adrenal medulla and represent the characteristics of ADRN-type NBLs. The cyan cluster had a wide range of TFs, including endothelial, stromal, and immune cells, but with medulla characteristics, and represented the characteristics of MES-type NBLs. TFs in the blue cluster without a network structure also had a wide range of features, including medulla, stromal and immune cells, and were characteristics of both the MES and ADRN types. Interestingly, while magenta and red were almost exclusively dedicated to the medulla, green also showed involvement with the endothelium, and this cluster showed the highest st4 and 4S classification performance. The green cluster, while located on the periphery of red, also has connections to parts of cyan, suggesting that it may serve as a bridge between the mutually exclusive red and cyan networks.

Previous studies have indicated that high SCP cell signatures are associated with a better NB prognosis [39]; however, based on our data, SCP signatures were only top-ranked in the cyan cluster. The cyan cluster did not significantly contribute to the distinction between st4 and 4S, suggesting that the SCP signature is not significant in the comparison of the properties of st4 and 4S. The cyan cluster was dominated by GO terms related to pigmentation, fat cell, B cell, insulin receptor signaling and smooth muscle. Moreover, adrenal cell signatures of this cluster were dominated by immune, stromal and endothelial TFs (see Additional file 5).

As the red and green clusters contained information that distinguished the two stages, st4 and 4S, some mechanisms controlled by the clusters would cause spontaneous regression of NBLs. Although the classification in our GCN graphs was consistent with some of the conventional clinical indicators, such as MYCN amplification, it did not appear to be consistent with other indices. This suggests that the genetic background and clinical indicators do not match. It is particularly noteworthy that some of the st4 tumor nodes resided near the 4S in the GCN graph drawn by the green cluster. This indicates that these st4 tumors have characteristics similar to those of 4S tumors.

HOX genes are not only involved in fetal organogenesis, but are also required for organ maintenance after birth and are also expressed in malignant tumor tissue. In malignant tumors, the expression of specific types of HOX genes is known to be related to malignancy and prognosis (see [44, 45]) and has some bearing on tumor character. Until now, studies of HOX genes in tumors have been limited to the abundance of individual gene expression and have not been systematically investigated. In this study, we showed that each group of HOX genes ABCD formed its own network in the co-expressed gene network. We also showed that the GSE49711 data, which was used for confirmation, is approximately reproducible. However, the HOXD cluster were not reproduced.

Although the reproducibility of the present results was verified using only one data set (GSE49711), it was confirmed that the combined clusters (red, green and magenta) and the cyan cluster appeared separately as a network structure, and it was reproduced that the combined cluster is deeply involved in the separation of st4 and 4S. On the other hand, the subnetwork structure of red surrounded by green and magenta was not reproduced. One reason for this is that, unlike the red and cyan clusters, which are strongly correlated, the green and magenta clusters are weakly connected, easily broken by noise and the change of cutoff values. Considering that the HOX genes are located in this weakly connected region, it may be necessary to eliminate the effects of noise or other factors to accurately capture the weak interactions between transcription factors.

Analysis of a large-scale population averages different features, such as GO, while analysis of a small-scale population, such as detailed clustering, loses the essential network structure. Therefore, it is important to extract an intermediate-scale population with common features while maintaining the essential structure. However, it is generally not easy to find the optimal intermediate-scale population for separating two groups. This paper argues that GTOM with RECODE is effective in finding such an intermediate-scale population.

## Limitations and future works

In this study, we confirmed the importance of the subnetworks that characterize 4 and 4S using GTOM among network analysis methods. It remains to be seen whether other network analysis methods can detect similar subnetworks.

In addition, in this study, we conducted a thorough network analysis on one set of NBL data, and only checked the reproducibility on the other set of data. Although we were able to obtain a certain degree of reproducibility, analysis of a larger number of data is an issue for the future.

We found the appropriate parameters in the view point of the GTOMscores and the SD of the number of nodes in clusters. Further study is needed on the validity of the evaluation formula for calculating the index for finding characteristic subnetworks from such GTOMscores and the search for better evaluation formulas.

The reproducibility of the results of the TARGET experiment was verified by GSE49711, but since there were differences in the data at the time of acquisition between the two, more accurate reproducibility verification is needed in the future. In particular, the intermediate subnetworks we focused on, such as green and magenta clusters, are located in sparsely connected regions, suggesting that the network structure may be significantly affected by noise.

If a method to regulate HOX genes in time series is invented, it may be possible to interfere with tumor growth. In addition, if a technology for single-cell RNAseq from a single tumor mass is developed, it will be possible to create a gene co-expression network of an individual tumor and plan a tailor-made therapy by looking at the gene map. Such treatment will be less damaging to the whole body than the current “total cell kill” therapy.

Finally, we suggest that this method may be versatile enough to detect subnetworks that are caused by differences between two different states produced by a large-scale network structure such as a gene network. Therefore, although the present experiments were conducted on NBL st4 and 4S, applying the method to other cases created by gene networks, and finding the characteristics of the original data or correlation networks for which the method is effective is also future works.

## Conclusion

This study proposed a classification method for st4 and 4S NBL using GCN analyses based on RNA sequencing data of TFs. Especially, the clustering method using GTOM was shown to be effective in finding a sub-network of TFs to classify st4 and 4S. We also showed that the combination with the RECODE method was more effective. This method of clustering through an intermediate-scale network may be useful as a way to extract factors that contribute to the separation of the two groups for multiple pieces of information such as gene expression levels. The characteristics of the sub-networks of TFs identified in this analysis were related to the NBL type (ADRN/MES) and mouse fetal adrenal cell signature. More detailed investigations of gene function based on these clusters will be required in the future. It is hoped that this analytical method will be used to recategorize NBL and the development of new therapies.

## Supplementary information

Additional file 1. A classification list of mouse fetal adrenal gland cells in [39].

**Additional file 2.** A result of GO analysis of each cluster.

**Additional file 3.** A list of NBL type (ADRN/MES) over TFs in our clusters.

**Additional file 4.** A list of mouse fetal adrenal cell gene signatures over TFs in our clusters.

**Additional file 5.** A list of the number of TFs belonging to each sub-group of fetal adrenal cells.

**Additional file 6.** Patients information.

**Additional file 7.** A Graph of several indices (e.g. the silhouette coefficient).

**Additional file 8.** Illustration of the projection onto a two-dimensional plane by tSNE (left) and UMAP (right) to the TARGET data with RECODE. The colors are the same as those of GTOM(2) with RECODE.

**Additional file 9.** A HOX gene placement in TARGET-NBL (after RECODE and GTOM analysis). HOX genes are indicated by square symbols. The color of each symbol indicates the cluster to which it belongs (cyan, red, magenta, green, blue). HOXA is marked in green, B in red, C in blue, and D in orange. Some parts of the graph are not shown for clarity. Figure A is with RECODE and Figure B is without RECODE.

**Additional file 10.** A HOX gene arrangement in GSE49711 (after RECODE and GTOM analysis). HOX genes are indicated by square symbols. The color of each symbol indicates the cluster to which it belongs (cyan, red, magenta, green, blue). HOXA is marked in green, B in red, C in blue, and D in orange. Some parts of the graph are not shown for clarity. Figure A is with RECODE enforcement and Figure B is without enforcement. The data used have been converted to TPM units to be consistent with the TARGET data.

## Data Availability

The dataset(s) supporting the conclusions of this article is(are) available in TARGET (https://www.cancer.gov/ccg/research/genome-sequencing/target) (phs000467.v1.p1.) and are downloaded via Genomic Data Commons (GDC) portal site (https://portal.gdc.cancer.gov/repository). About the preprocessing of the original data, refer to the following URL (https://docs.gdc.cancer.gov/Data/Bioinformatics_Pipelines/Expression_mRNA_Pipeline/#mrna-expression-transformation). In short, the primary counting data is generated by STAR. Following alignment, the raw counts files produced by STAR are transformed to commonly used count unit (TPM). GENCODE v36 was used for gene annotation. Clinical data are downloaded via GDC portal site (https://portal.gdc.cancer.gov/) as TARGET_NBL_ClinicalData_Discovery_20230523.xlsx < UUID: f5bfc8a5-b903-4418-b633-62234388a635> (see Additional file 6).

## Abbreviations

NBL: Neuroblastoma
st4: Stage 4
4S: Special stage 4
GCN: Gene coexpression network
GCNA: GCN analysis
scRNA-seq: Single-cell RNA sequencing
GO: Gene ontology
TOM: Topological overlap measure
GTOM: Generalized TOM
TF: Transcription factor
MKI: Mitotic-Karyorrhectic Index
FDR: False discovery rate
SCP: Schwan Cell Precursor
RECODE: Resolution of the curse of dimensionality
PCA: principal component analysis
Q3: Third quarter
tSNE: t-distributed stochastic neighbor embedding
UMAP: Uniform manifold approximation and projection
MES: Mesenchymal
ADRN: Adrenergic
