# Supplementary figures and images for: Fine construction of gene coexpression network analysis using GTOM and RECODE detected a critical module of neuroblastoma stages 4 and 4S
Source: Hereditas. 2024 Nov 14;161:44. doi: 10.1186/s41065-024-00342-y (PMC11562103; doi:10.1186/s41065-024-00342-y)

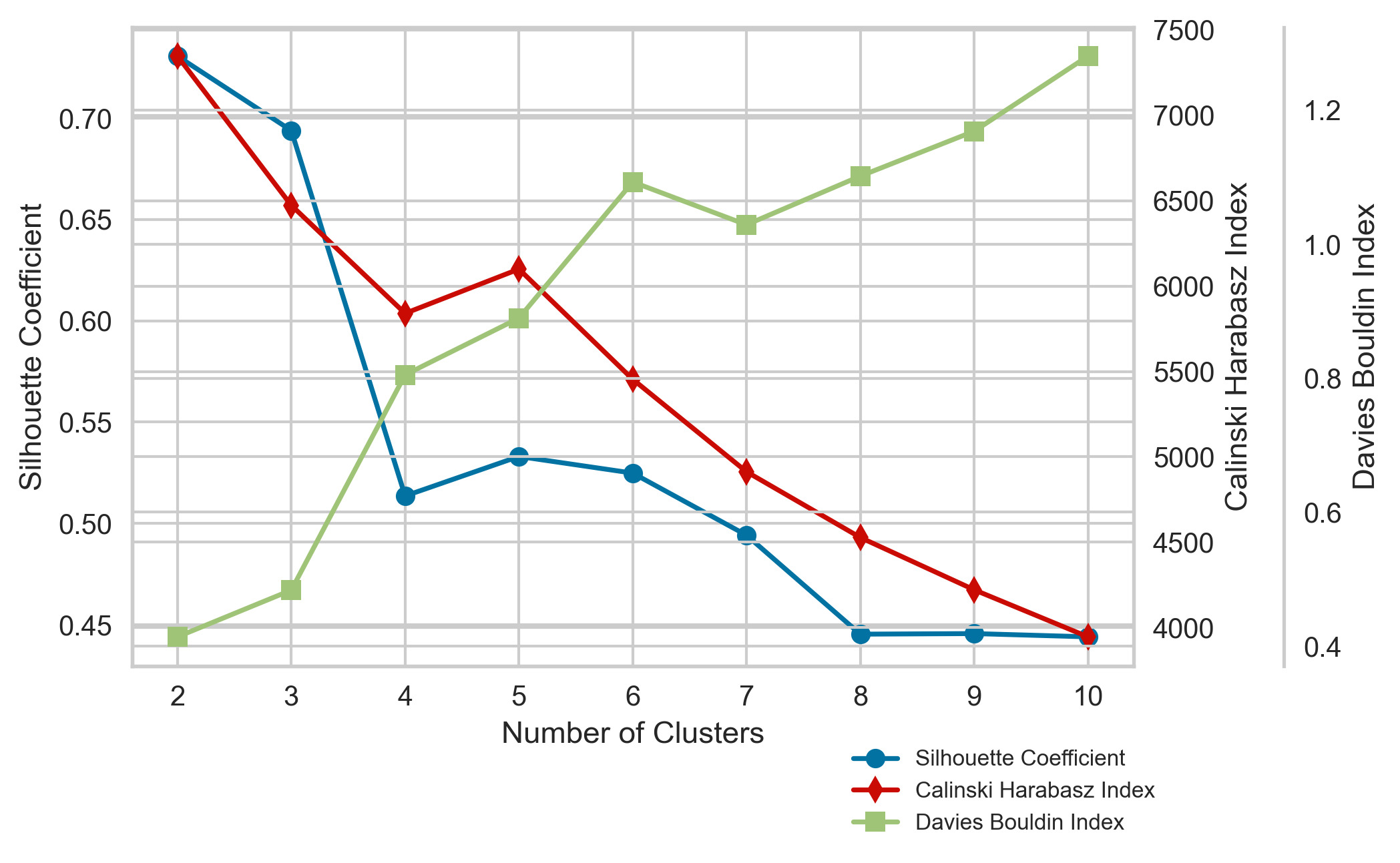

Supplement: Supplementary file 7 — Additional file 7. A Graph of several indices (e.g. the silhouette coefficient). [file 41065_2024_342_MOESM7_ESM.jpg]

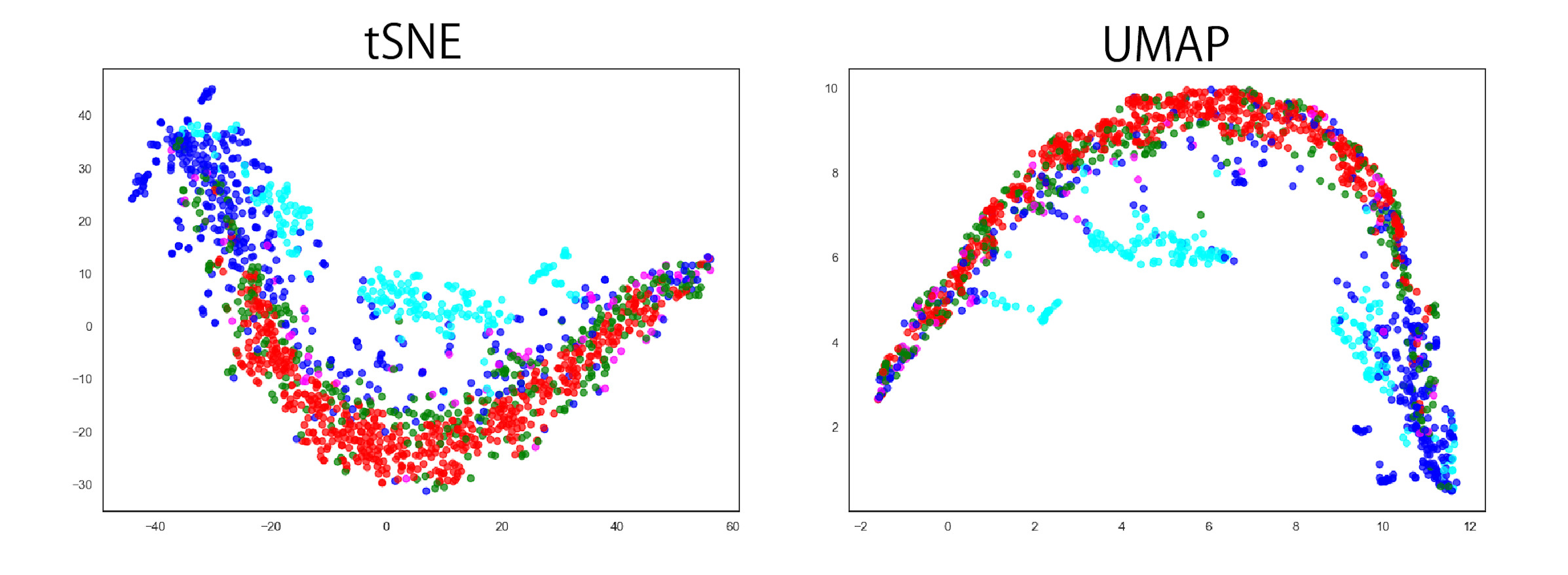

Supplement: Supplementary file 8 — Additional file 8. Illustration of the projection onto a two-dimensional plane by tSNE (left) and UMAP (right) to the TARGET data with RECODE. The colors are the same as those of GTOM(2) with RECODE. [file 41065_2024_342_MOESM8_ESM.jpg]

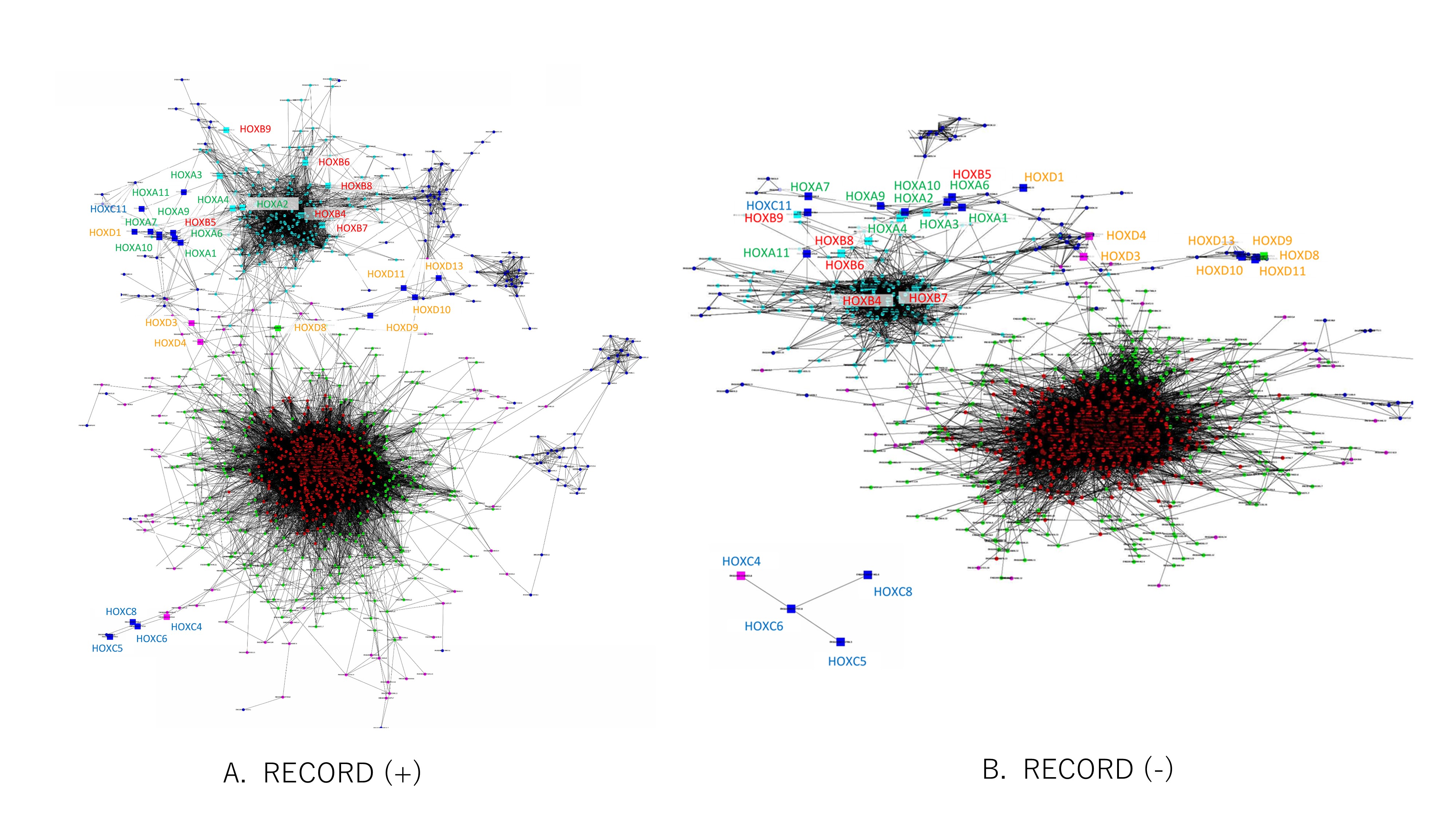

Supplement: Supplementary file 9 — Additional file 9. A HOX gene placement in TARGET-NBL (after RECODE and GTOM analysis). HOX genes are indicated by square symbols. The color of each symbol indicates the cluster to which it belongs (cyan, red, magenta, green, blue). HOXA is marked in green, B in red, C in blue, and D in orange. Some parts of the graph are not shown for clarity. Figure A is with RECODE and Figure B is without RECODE. [file 41065_2024_342_MOESM9_ESM.jpg]

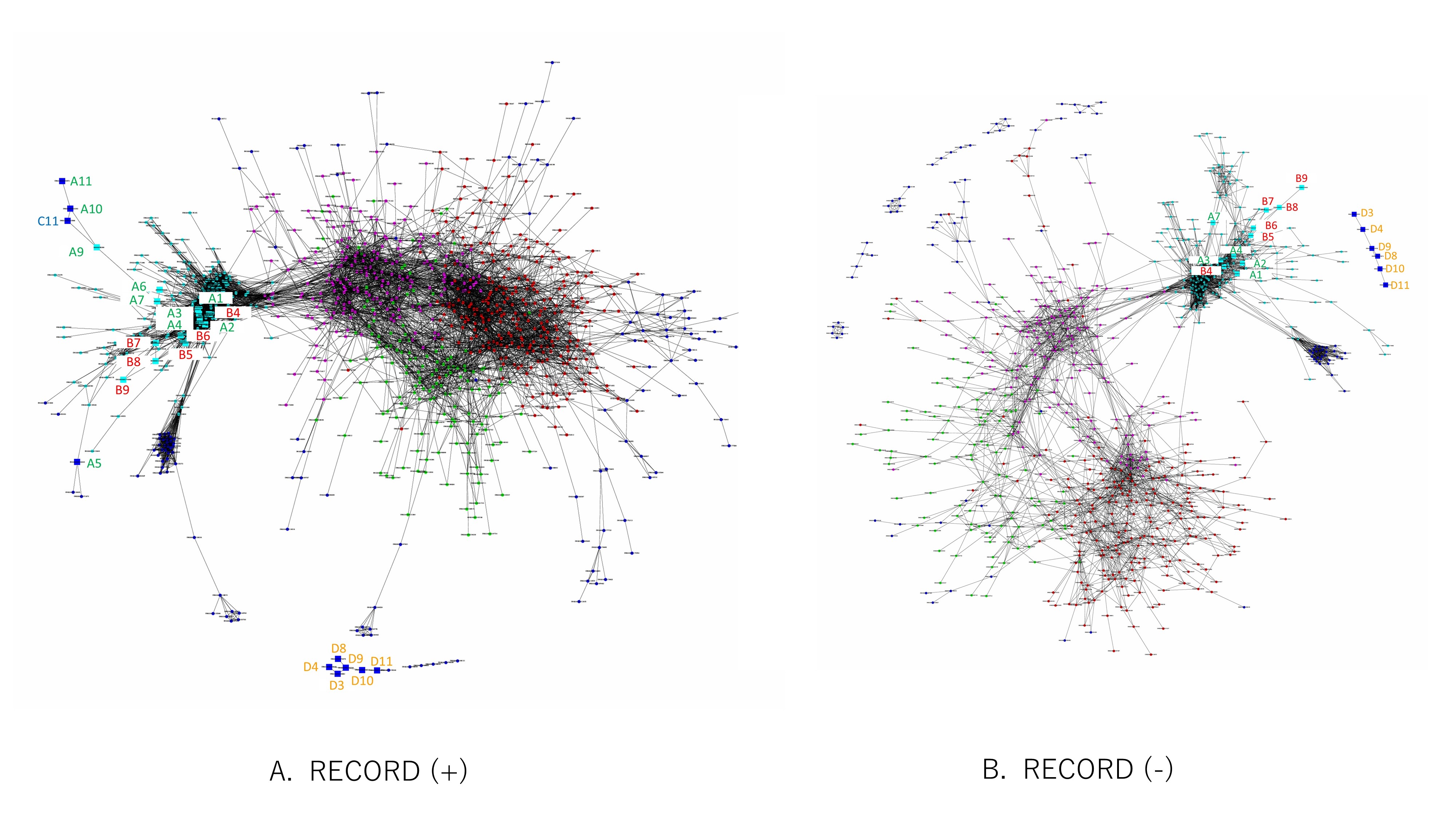

Supplement: Supplementary file 10 — Additional file 10. A HOX gene arrangement in GSE49711 (after RECODE and GTOM analysis). HOX genes are indicated by square symbols. The color of each symbol indicates the cluster to which it belongs (cyan, red, magenta, green, blue). HOXA is marked in green, B in red, C in blue, and D in orange. Some parts of the graph are not shown for clarity. Figure A is with RECODE enforcement and Figure B is without enforcement. The data used have been converted to TPM units to be consistent with the TARGET data. [file 41065_2024_342_MOESM10_ESM.jpg]
